# Recipient contributions to primary graft dysfunction in heart transplantation

**DOI:** 10.3389/fcvm.2026.1984203

**Published:** 2026-10-27

**Authors:** Ahmed El-Hamad, Nitish K. Dhingra, Vivek Rao

**Affiliations:** 1 Temerty Faculty of Medicine, University of Toronto, Toronto, ON, Canada; 2 Division of Cardiac Surgery, University of Toronto, Toronto, ON, Canada; 3 Peter Munk Cardiac Center, University Health Network, Toronto, ON, Canada

**Keywords:** amiodarone, early graft dysfunction, heart transplant, left ventricular assist device, mechanical circulatory support, primary graft dysfunction, recipient risk factors

## Abstract

Primary graft dysfunction (PGD) remains the leading cause of early mortality post heart transplantation (HT). Across several international and national registries, the incidence of PGD has continued to increase. The mechanistic contributions of donor and procedural insults, namely brain or circulatory death, ischemic exposure, and ischemia-reperfusion injury, have been consistently emphasized across various reports. As the surgical techniques and procurement strategies within the current era of HT continue to improve, the net of recipients broadens and includes more high-risk, frail, and comorbid patients. This review therefore aims to explore the recipient contributions of PGD across four domains: hemodynamics and use of mechanical circulatory support, preoperative medical therapy, end-organ dysfunction, and recipient-driven operative characteristics. For each domain, we describe its incidence, the predictor's underlying mechanism, and its contribution to the recipient's biology. Durable LVAD support, pre-transplant ECMO, amiodarone exposure, renal dysfunction, and prior sternotomy emerge as consistently reported independent predictors of PGD, whereas findings for temporary VAD and IABP remain inconsistent. These exposures provide distinct pathways to generate a hostile recipient environment characterized by a primed innate immune system, systemic inflammation, hemodynamic burden, vasomotor dysfunction, multi-organ dysfunction, and operative burden. However, no interventional study has yet to examine the effect of modifying recipient risk factors on PGD development; thereby, existing evidence are derived from observational studies implying association rather than causation. Bridging the gap between recipient risk factors and biologic contributions to PGD may unveil areas for recipient optimization, ultimately improving HT outcomes.

## 1 Introduction

Heart transplantation (HT) remains widely accepted as the cornerstone treatment for advanced heart failure, with increasing rates supported by enhancements in preservation strategies and expanded donor pools (1–3). Despite contemporary advancements and improvements in survival, HT remains adversely impacted by primary graft dysfunction (PGD).

PGD, defined as an acute allograft failure to meet circulatory requirements in the absence of an identifiable cause or complication within the first 24-hours post-transplant period has various implications on recovery, multiorgan function, and survival, ultimately serving as a leading contributor to early post-transplant mortality (4–7). In an analysis of 2,746 patients from the International Consortium on PGD across 10 centers in the United States, Canada, and Europe, severe PGD occurred in 7.8% of recipients with a one-year survival of 75.7% vs 94.4% in those with and without severe PGD, respectively (8). Similarly, a contemporary analysis of the United Network for Organ Sharing (UNOS) database reported severe PGD in 6.6% of 5,097 patients and a lower three-month survival of 74.4% vs 96.8% in those without severe PGD (9).

The burden of PGD is not diminishing. Overall incidence of severe PGD has significantly increased across international and national registries (8, 10). Reducing the burden requires addressing the pathophysiology across the entire continuum of risk factors. Donor risk factors have consistently been described across various reports including age (6, 8), female sex (11), and donation after circulatory death (DCD) (9). Procedural factors, namely blood transfusions (12), prolonged ischemic time (8, 9), and cardiopulmonary bypass (CPB) time (13), are well established.

While associations of recipient risk factors with PGD, such as prior sternotomy (9, 14), durable LVAD support (8, 9, 15), and amiodarone therapy (16, 17) are commonly reported, their contribution to the underlying pathophysiology of PGD remains less well-studied.

In an era of advancements in surgical techniques and donor heart preservation strategies, the evolving recipient populations with greater use of mechanical circulatory support (MCS), more multi-organ failure, higher frailty, and cumulative preoperative drug exposures (8, 18), likely contributes to the rising incidence of severe PGD. The relative contribution of donor and recipient risk matching was examined in an International Society for Heart and Lung Transplantation (ISHLT) registry study from 2000 to 2018, where donor risk was quantified through the Donor Utilization Score and recipients' risks was determined through the IMPACT score comprising 12 recipient variables (19, 20). In North American recipients, one-year graft failure was highest when pairing high-risk recipients with high-risk donors (13.1%, 95% CI, 10.7%-13.9%) and lowest among low-risk recipients with low-risk donors (7.4%, 95% CI 6.8%-8.0%) (20). Interestingly, matching of high-risk recipients with low-risk donors was associated with higher rates of graft failure (11.4%, 95%CI 10.7%-12.2%), compared to low-risk recipients with high-risk donors (9.0%, 95% CI, 8.3%-9.7%) (20). Irrespective of donor risk, the presence of high-risk recipients conferred an increase of nearly four percentage points in one-year graft failure rates, while high-risk donors only increased rates under two percentage points. These findings suggest that recipient risk serves as a significant driving force behind post-transplant outcomes. The components of recipient risk therefore warrant closer characterization to understand and optimize their effects on PGD.

Therefore, this review aims to describe the underlying contribution of recipient characteristics – several of which comprise the IMPACT score described above – covering hemodynamics and MCS, preoperative medical therapy, end-organ function, and perioperative surgical factors, and examine their pathophysiologic effect on developing PGD.

### 1.1 Definition of PGD

The 2014 ISHLT consensus on PGD reached a universal definition with aims to better define, diagnose, and manage PGD (21). Where a discernible cause for graft dysfunction is present, such as hyperacute rejection, pulmonary hypertension, and intraoperative surgical complication, the syndrome is termed secondary graft dysfunction rather than PGD (21). PGD is further divided into ventricular involvement: PGD-LV refers to left ventricular or biventricular failure, while PGD-RV refers to isolated right ventricle involvement. In terms of severity, PGD-LV includes mild, moderate, and severe, each reflecting hemodynamic variables, echocardiography results, inotropic support, and need for mechanical circulatory support (21). Severe PGD is distinguishable from mild-moderate PGD, requiring dependence on left or biventricular mechanical support such as ECMO or LVAD, explicitly excluding a requirement for IABP alone (21). In contrast, PGD-RV does not incorporate a severity scale, with diagnosis reflecting hemodynamic variables and the need for RVAD support.

The contemporary 2024 ISHLT consensus on graft dysfunction now provides a newly updated definition (22). Dysfunction of unknown cause occurring < 24 hours post-HT is now termed EGD, while dysfunction occurring at 24-72 hours post-HT is termed subsequent early graft dysfunction (sEGD) (22). EGD is likewise divided into ventricular involvement and replaces the three-grade severity with two categories, non-severe and severe, per ventricular subtype. Both severe and non-severe EGD require a reduced ejection fraction and hemodynamic compromise necessitating inotropic support; severe EGD, however, additionally requires left or biventricular mechanical support such as VA-ECMO, LVAD, BiVAD, and now including intra-aortic balloon pump (IABP) which was explicitly excluded in the 2014 definition (21, 22). Severity is therefore determined by echocardiography results and the need to initiate mechanical circulatory support (MCS) rather than inotrope scoring (22). Further, severe EGD is distinguished by timing of presentation as either immediate presentation defined as failure to wean from CPB (EGD-LVip) or delayed presentation occurring after successful weaning from bypass (EGD-LVdp) (22). The proposed inclusion of IABP support as diagnostic for severe-EGD has raised questions regarding its prognostic implications. Among 7,239 HT recipients in two contemporary registries, recipients classified as severe EGD solely by IABP had significantly lower 1-year mortality compared to those classified by the 2014 criteria (12.1% vs 36.3% in International PGD Consortium database; 9.6% vs 27.2% in UNOS) (23).

Accordingly, the current evidence base, including studies cited in this review, predate the 2024 updated definition, and refers to the 2014 PGD criteria. For consistency with the current literature, PGD is used throughout this review, with EGD reserved for the updated framework and studies that applied the updated criteria, with the recognition that future studies will likely adopt the EGD terminology.

### 1.2 PGD, secondary graft dysfunction, and vasoplegia

Although often layered with PGD, secondary graft dysfunction and vasoplegia are distinct clinical entities. Secondary graft dysfunction, as previously described, refers to graft dysfunction due to a discernible cause according to the 2014 ISHLT definition and updated framework for EGD (21, 22). Vasoplegia, on the other hand, is characterized as a state of low systemic vascular resistance (SVR) despite preserved or elevated cardiac index and refractory to fluids or vasopressors (24). Vasoplegia often co-exists with PGD with a recent pooled analysis reporting vasoplegia in 28.7% of HT recipients (22, 25). Likewise, these syndromes share similar pre-transplant risk factors and pathophysiological pathways: the inflammatory environment in PGD may lower SVR predisposing to vasoplegia, while the use of vasopressors for refractory vasoplegia may contribute to PGD development (22). While these entities often overlap, we herein classify each recipient-driven mechanistic pathway as a factor associated with PGD, a plausible but unvalidated contributor to EGD, a potential cause of secondary graft dysfunction, or a mechanism more consistent with vasoplegia, Table 1.

**Table 1:** Associations of recipient risk factors to graft dysfunction: proposed mechanisms, nature of supporting evidence, and underlying classification. AOR, adjusted odds ratio; CF-LVAD, continuous-flow left ventricular assist device; CI, confidence interval; CPB, cardiopulmonary bypass; CPRA-LS, calculated panel reactive antibody, locus-specific; CVP, central venous pressure; ECMO, extracorporeal membrane oxygenation; HLA, human leukocyte antigen; HR, hazard ratio; HT, heart transplantation; IABP, intra-aortic balloon pump; IgG, immunoglobulin G; IRI, ischemia-reperfusion injury; LVAD, left ventricular assist device; MCS, mechanical circulatory support; MRA, mineralocorticoid receptor antagonist; NR, not reported; OR, odds ratio; PCWP, pulmonary capillary wedge pressure; PRBC, packed red blood cells; RAS, renin angiotensin system; RV, right ventricle; T3, triiodothyronine; T4, thyroxine; VAD, ventricular assist device. .

Recipient risk factor	Proposed mechanism	Nature of supporting evidence	Key Limitations	Classification
Durable LVAD	RV dysfunction and RHF post-LVAD may cause end-organ dysfunction, venous congestion, and a primed inflammatory response	Higher CVP:PCWP ratio in severe PGD (0.70 vs 0.54; *P* = .004) (62); Severe PGD in 23.8% of LVAD-supported patients with late RHF vs 6.67% without RHF (*P* = .012) (65); Post-LVAD RHF independently associated with a four-fold increased risk of PGD (OR 3.91; 95% CI, 1.61–9.68; *P* = .003) (66).	The association may reflect the clinical instability of this population; direct mechanistic evidence describing a clinical pathway does not yet exist and remains unvalidated	PGD; unvalidated for EGD
	Host immune activation of HLA and non-HLA antibodies	Cohort of 132 CF-LVADs patients had 23% new allosensitization (69); peak CPRA-LS for HLA-A OR 1.14 (95% CI, 1.03-1.26); IgG reactivity associated with PGD HR 1.88 (95% CI, 1.23–2.87) (75)	Direct binding of antibodies to allograft not demonstrated	PGD; unvalidated for EGD
	Non-pulsatile flow induces arterial remodeling.	Increase in post-HT aortic vascular stiffness in CF-LVAD patients (80)	No explicit link to PGD established	No reported association to PGD; unvalidated for EGD
Temporary MCS	Not uniform across devices; recipients sustain cardiogenic shock, systemic inflammation, high metabolic demands, and multiorgan hypoperfusion	Pre-transplant ECMO independently associated with severe PGD (AOR, 2.55; 95% CI, 1.63–3.98; *P* < .001) and IABP and temporary VAD not significant (9); single-center analysis reporting IABP as the opposite (83)	Mechanism has not been tested in HT recipients	PGD; unvalidated for EGD
Preoperative mechanical support	May be best interpreted as a marker of the critically ill, decompensated, high-risk recipient	Univariable association (OR, 4.34; 95% CI, 1.96–9.61; *P* < 0.001) not retained on multivariable modelling (86); no difference between severe PGD and control (2.9% vs 2.2%; *P* = .37) (9)	No mechanistic study demonstrating direct link to PGD; often concurrent with the use of temporary MCS	PGD; unvalidated for EGD
Amiodarone	Persistence of amiodarone in the allograft given its lipophilic nature; Negative chronotropic/inotropic effects on calcium channel inhibition and *β*-receptor blockade	Single-center cohort of 183 patients found no associations (91); strongest predictor in a recent meta-analysis (OR, 4.38; 95% CI, 2.48-7.75); dose-dependent response supported (17); continued therapy until HT (AOR, 1.63; 95% CI, 1.31-2.01) (16)	Concentration of amiodarone in allograft has yet to be related to PGD; evidence remains conflicted with opposing single-center analysis	PGD; unvalidated for EGD
	Synergistic effect with β-blockers on the catecholamine-depleted β-adrenergic receptors previously described in brain death	Combination of amiodarone + β-blockers carried higher odds of severe PGD (OR, 12.14; 95% CI 5.03-29.29; *P* < .001) and retained on multivariable modelling (12)	Mechanism of synergy has not been proven and remains speculative	PGD; unvalidated for EGD
RAS inhibitors with MRA	Accumulation of vasodilatory peptides, such as bradykinin, a potent vasodilator that potentiates during CPB	Combination of drugs associated with severe PGD (OR, 3.33; 95% CI, 1.57-7.08; *P* = .002) (12)	Single-center analysis that was absent from 2026 meta-analysis; bradykinin finding may reflect vasoplegic syndrome rather than PGD	Mechanism consistent with vasoplegia
Thyroid hormone therapy	Cardioprotective effects through enhanced myocardial contractility via calcium reuptake and mitochondrial protection against IRI	Decreased free T4 levels independently associated with PGD (OR, 6.49; 95% CI, 2.26-18.61; *P* = .001) (100); T3 supplementation did not reduce PGD incidence (105)	Mechanism largely extrapolated from experimental findings; randomized trial currently underway	PGD; unvalidated for EGD
Renal dysfunction	Cardiorenal congestion: RV dysfunction where elevated right-sided pressures retrogradely propagate and impair the renal perfusion gradient	Renal replacement therapy at HT as a severe PGD predictor (OR, 2.41; 95% CI, 1.31–4.43) (8); dose-dependent relationship with creatinine levels (4)	Pre-transplant creatinine levels is a surrogate for venous congestion and may not serve as a direct measure of renal dysfunction	PGD; unvalidated for EGD
	Inflammation and oxidative stress associated with renal insufficiency (type III cardiorenal syndrome)	NR	Mechanism extrapolated from cardiorenal literature and yet to be examined	No reported association with PGD
Hepatic dysfunction	Elevated bilirubin, a marker for hepatic congestion, may be associated with severe PGD	Well-known predictor in general cardiac surgery (111); Elevated bilirubin associated with PGD in unadjusted modelling (OR, 1.11; 95% CI, 1.02–1.21; *P* = .012) yet null in multivariable modelling (86)	Evidence remains limited to general cardiac surgery outcomes with no mechanistic literature on transplant-specific populations	PGD; unvalidated for EGD
Prior sternotomy	Operative complexity attributable to mediastinal adhesions increasing CPB and ischemic time	Independent predictor of severe PGD (AOR, 1.83; 95% CI, 1.33–2.51; *P* < .001) (9); non-LVAD sternotomy was also an independent predictor (OR, 1.96; 95% CI, 1.01-3.81) (64)	Effect may be indirect as largely mediated by downstream operative exposures; recipients with prior sternotomy also largely differ at baseline presenting potential confounders	PGD; unvalidated for EGD; potential cause of secondary graft dysfunction if identifiable
	Greater bleeding necessitating increased transfusions which may overload RV function, generate inflammatory mediators, worsen IRI.	Different products associated with severe PGD: PRBC OR 1.18, platelets OR 1.45, plasma OR 1.13, cryoprecipitate OR 1.04 (12)	Mechanisms largely extrapolated from general cardiac surgery and transfusion literature rather than transplant-specific settings	PGD; unvalidated for EGD; potential cause of secondary graft dysfunction if identifiable
Cardiopulmonary bypass time	Interaction between recipient blood and synthetic CPB circuit inducing systemic inflammation: cytokine release, oxygen-free radical formation, coagulation dysfunction, vasoplegia	Longer CPB time associated with PGD with an AOR of 1.75 per 60-minute increase (95% CI, 1.44–2.14; *P* < .0001) (13)	Directionality cannot be confirmed; CPB time may be a consequence of PGD rather than a predictor	PGD; unvalidated for EGD; inflammatory burden consistent with vasoplegia

## 2 Overview of PGD pathophysiology

The donor heart sustains a multitude of insults at defined time points in the transplant process including donor brain or circulatory death, periods of cold and/or warm ischemia and ischemic-reperfusion injury (5, 21, 26). Recipient factors may further exacerbate ischemic reperfusion injury and compromise donor heart function after reperfusion (21). PGD therefore likely emerges from the summation of these factors, framing the pathophysiology as a multi-hit syndrome, Figure 1. For the purposes of this paper, we will focus predominantly on recipient contributions to ischemic reperfusion injury and the pathophysiology of PGD.

**Figure 1:**
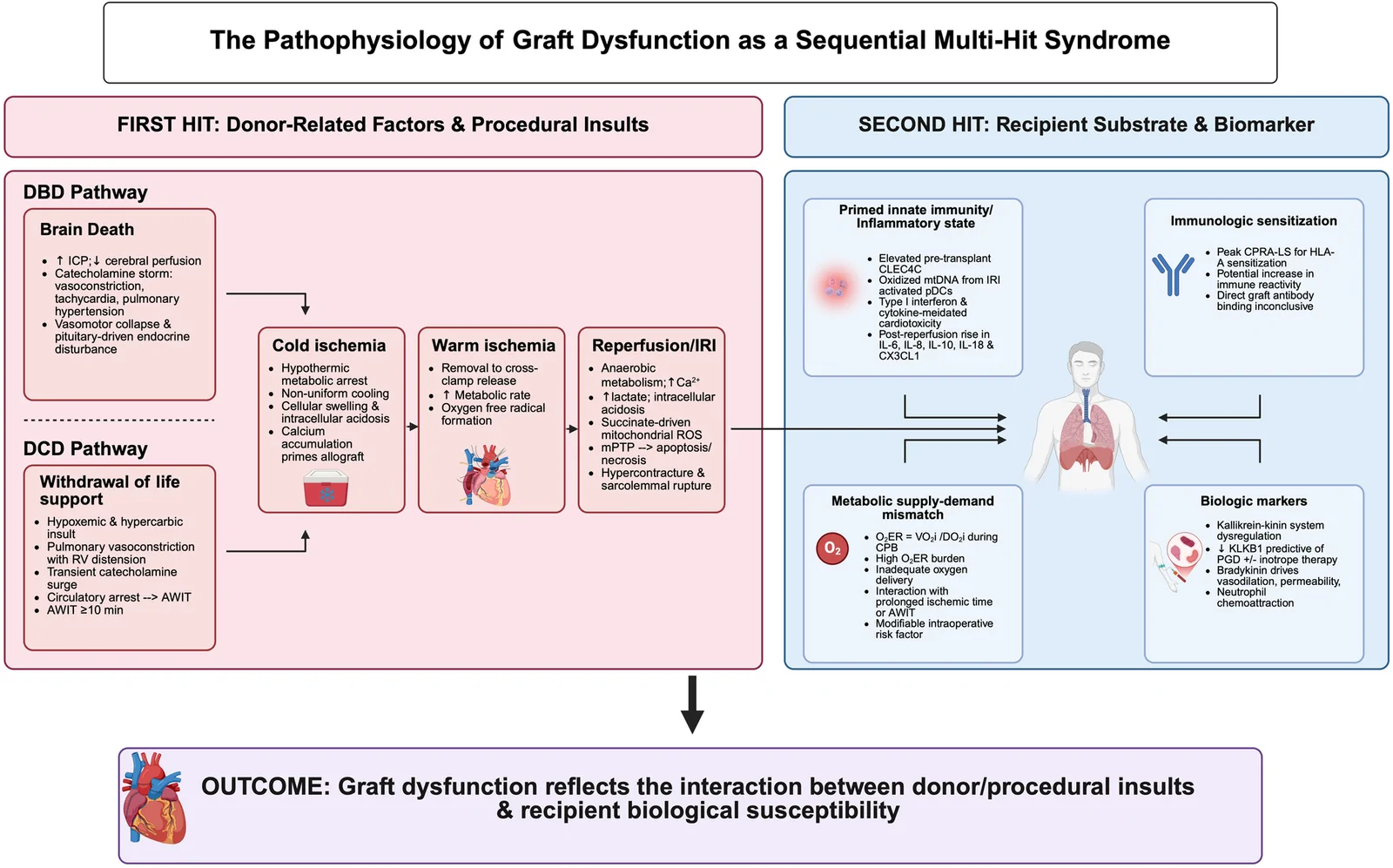
The pathophysiology of graft dysfunction framed as a mechanistic sequential multi-hit syndrome. AWIT, asystolic warm ischemic time; CLEC4C, C-type lectin domain family 4 member C; CPRA-LS, calculated panel reactive antibody, locus specific; HLA-A, human leukocyte antigen type A; ICP, intracranial pressure; IRI, ischemia-reperfusion injury; KLKB1, plasma kallikrein; mPTP, mitochondrial permeability transition pore; mtDNA, mitochondrial DNA; pDCs, plasmacytoid dendritic cells; PGD, primary graft dysfunction; ROS, reactive oxygen species.

### 2.1 Donor-related factors and procedural insults

Brainstem death marks the first insult in the transplant process. Regardless of the cause of death, the final common pathway is elevated intracranial pressures (ICP) and reduced cerebral perfusion pressure (CPP) (26). A persistent rise in ICP can cause brain herniation resulting in pontine ischemia (26, 27). A catecholamine-storm ensues causing intense vasoconstriction, tachycardia, and pulmonary hypertension, all contributing to increased afterload and ultimate myocardial ischemia (5, 28). Vasomotor collapse follows due to loss of spinal sympathetic tone resulting in unopposed vasodilatation and impaired cardiac output (27), concurrent pituitary ischemia may cause endocrine disturbances such as diabetes insipidus, hyperglycemia, and hypothermia, which further destabilises the donor (29). The graft is subsequently exposed to preservation injury, namely cold ischemia. Conventional cold storage induces hypothermic metabolic arrest, but cooling is rarely uniform, exposing hearts to fluctuations particularly those of older donor with left ventricular hypertrophy that are susceptible to uneven cooling and ischemic injury (5, 30–32). Prolonged cold ischemic time may lead to cellular swelling, intracellular acidosis, and ultimately intracellular calcium accumulation predisposing to ischemia-reperfusion injury (33).

Warm ischemic time, defined as the time from removal of the heart from the cold storage to removal of the recipient aortic cross clamp (5), augments metabolic rate and the formation of oxygen-free radicals causing cellular damage (33), and has been associated with reduced survival and increased 30-day mortality (34, 35).

Hearts donated after circulatory death (DCD) follow a parallel pathway but do not experience a catecholamine storm. Following withdrawal of life support, the DCD heart sustains hypoxemic and hypercarbic insults, pulmonary vasoconstriction, and right ventricular (RV) distension, followed by a transient catecholamine surge preceding circulatory arrest (36, 37). A warm ischemic state ensues with prolongation leading to similar ischemia-reperfusion injury upon reperfusion (36). DCD independently predicted severe PGD in a UNOS cohort (AOR, 2.13; *P* < .001) (9); likewise among 143 DCD recipients, asystolic warm ischemic time (AWIT) ≥ 10 minutes independently increases the risk of severe PGD (OR, 2.99; 95% CI, 1.29-7.61; *P* = .01) (38), appearing to be a significant contributor of PGD in DCD transplantation.

### 2.2 Ischemia-reperfusion injury

Ischemia-reperfusion injury (IRI) is the foundational and traditionally accepted pathophysiological mechanism of PGD, with the magnitude of its injury set by total ischemic time. In the ischemic state, the limited oxygen supply switches the graft to an anaerobic metabolism which results in the accumulation of lactate and intracellular acidosis (39). The accumulated protons activate sodium-hydrogen ion exchangers that promote hydrogen release for sodium entry; sodium-calcium exchangers then function to remove sodium, ultimately leading to intracellular calcium overload (39, 40). In parallel, the accumulation of the citric acid cycle intermediate succinate arises due to the reversal of succinate dehydrogenase (41). The combination of these events establishes the substrate for the injury that follows.

The release of the recipient cross-clamp marks the onset of reperfusion, where oxygenated blood reperfuses the previously oxygen-deprived heart, leading to calcium overload and release of oxygen free radicals (40). Upon reperfusion, succinate dehydrogenase serves to rapidly re-oxidise the accumulated succinate, generating an extensive burst of mitochondrial reactive oxygen species proportional to the quantity accumulated during ischemia (41). Together, these activate the non-specific mitochondrial permeability transition pore which triggers a cascade of cell-death pathways; apoptosis through release of cytochrome C, and cell swelling leading to necrotic cell death (42).

A second mechanism of IRI occurs, known as hypercontracture-mediated sarcolemma rupture. During the ischemic state, the low cytosolic ATP is rapidly depleted resulting in myofibrillar shortening due to actin-myosin cross-bridges remaining in a fixed state (43). The sustained contracture leads to detectable cytoskeletal defects and increased cardiomyocyte fragility; however, this is reversible with early reperfusion (44). Upon reperfusion, ATP levels are restored and the presence of calcium overload results in hypercontracture leading to greater myofibrillar shortening, cytoskeletal damage, and a marked rise in ventricular wall-stiffness (5). Due to the impaired sodium-calcium exchangers, and degradation of structural proteins (45, 46), sarcolemmal rupture soon follows, and may propagate to adjacent cardiomyocytes through sodium influx via gap junctions (47). Further, histological analysis of reperfused myocardium reveals contraction band necrosis, supporting this mechanism of injury (48, 49).

### 2.3 The recipient substrate and biomarkers: the second hit

The recipient contribution to PGD has been conventionally described in terms of clinical risk factors; however, the biological setting those factors produce has received less attention. The hostile environment, illustrated by biomarkers in the pre-transplant recipient serum, thereby serves as the “second hit” to which the allograft is exposed.

Pre-transplant recipients house a primed innate immune system potentially leading to subsequent graft failure. In a cohort of 219 recipients, Truby et al. identified the CLEC4C protein among 354 profiled proteins to be associated with PGD (OR, 3.04; 95% CI, 1.74-5.82; *P* = .00028) (50). Importantly, the predictive performance of CLEC4C in combination with the covariates outperformed the RADIAL score (AUC 0.69 vs 0.55), although this was demonstrated in a single-center validation set of 88 recipients. CLEC4C serves as a surface marker for plasmacytoid dendritic cells (pDCs) which are primarily involved in the production of type I interferons and pro-inflammatory cytokines such as TNF and IL-6, particularly in response to oxidized mitochondria (mtDNA) (51). The elevated CLEC4C expression thereby proposes a plausible biological model for the development of PGD, by which the elevated mtDNA released during ischemia and ischemia-reperfusion injury activate the pDCs causing high interferon and inflammatory-mediated cardiotoxicity (52). Moreover, Giangreco et al.'s proteomic evaluation of 88 pre-transplant microvesicle serum samples identified decreased levels of plasma kallikrein (KLKB1) as the most predictive factor for PGD independently and in combination with pre-transplant inotrope therapy to reach an AUROC of 0.71 with NPV of 0.92-0.96 (53). KLKB1 serves as a key regulator in the inflammatory response of the kallikrein-kinin-system (KKS) through the conversion of kininogen into bradykinin, thereby stimulating the release of nitric oxide and prostacyclin causing significant vasodilation, vascular permeability, and neutrophil chemoattraction (53–55). The reported association of reduced plasma KLKB1 and increased KKS activity is not paradoxical: in sepsis, a markedly inflammatory state, increased KKS activity is associated with lower plasma KLKB1 attributed to consumption (53, 56). Whether this directionality extends to HT has not been established. To further characterize if the inflammatory risk for PGD precedes transplantation, Holmström et al. found that among 74 HT recipients, preoperative donor and recipient cytokines failed to predict PGD, while severe PGD, unlike mild-moderate, was associated with post-reperfusion rise in IL-6, IL-8, IL-10, IL-18 and CX3CL1 (57). Whether this cytokine rise during reperfusion is attributable to the recipient predisposition, or the magnitude of ischemic insult, remains uncertain.

Recipient immunological priming has also been identified prior to transplant. Han et al. analyzed 596 recipients across two centers and found peak calculated panel reactive antibody locus specific (CPRA-LS) for HLA-A was associated with increased severity of PGD, which was retained with multivariable modelling (OR, 1.14; 95% CI, 1.03-1.26; *P* = .011) (58). While promising, direct antibody binding with the graft was not demonstrated, thereby confounding cannot be excluded.

Recipients' intraoperative metabolic adequacy has been quantified using the oxygen extraction ratio burden (O_2_ER), defined as the ratio of indexed O_2_ consumption to delivery (VO_2_i/DO_2_i) during CPB and may serve as an indicator of adequate tissue oxygenation (59, 60). Prolonged allograft ischemic time predisposes the donor heart to IRI, an established risk factor for severe PGD (9, 59); unlike donor-related exposures, however, O_2_ER offers a practical and immediately measurable method of optimizing metabolic needs intraoperatively (59). Among 373 HT recipients, patients with prolonged ischemia and high O_2_ER burden (defined as more than 25% of bypass time spent above O_2_ER of 0.25) sustained the highest incidence of severe PGD at 14.6%, compared with 3.6%, 3.3%, and 3.7% in short ischemia/low burden, short ischemia/high burden, and prolonged ischemia/low burden, respectively (*P* = .002); one-year all-cause mortality was distributed similarly (13.5% vs 2.7%, 2.2%, and 4.9%; *P* = .005). Within the prolonged ischemia subgroup, each 5% increase in CPB time spent over O_2_ER of 0.25 increased odds of severe PGD (OR, 1.16; 95% CI, 1.04–1.34; *P* = .005), while low O_2_ER was associated with lower odds of severe PGD (OR: 0.21, 95% CI: 0.07-0.54; *P* < .001), suggesting an O_2_ER-related risk independent of ischemic time (59). A parallel analysis of 112 DCD recipients recovered using thoracoabdominal normothermic regional perfusion, demonstrated similar findings where prolonged AWIT and high O_2_ER (defined as >0.20) had severe PGD occur in 22.2% of recipients, compared with 8.6% in the short AWIT/low O_2_ER group, and none in short AWIT/high O_2_ER and prolonged AWIT/low O_2_ER (*P* = .005). Notably, within the prolonged AWIT group, lower O_2_ER burden was associated with reduced odds of severe PGD (OR 0.09, 95% CI 0.07-0.87; *P* = .02) (61). While these results remain limited to single-center observational cohorts and are hypothesis-generating, they show a potential association of O_2_ER on PGD development suggesting that when recipient oxygen delivery is inadequate, ischemic injury may translate into graft dysfunction; given the modifiable nature of this factor, further investigation is warranted.

Although these studies are derived from single-center cohorts and different platforms, taken together they converge on immune and inflammatory activation in the pre-transplant recipient. While many of these results are hypothesis-generating, they each provide relevant insights into the recipient environment that can contribute to PGD independently of allograft-related factors. The sections that follow discuss the underlying basis on how the known clinical exposures generate this multifaceted recipient environment, Figure 2.

**Figure 2:**
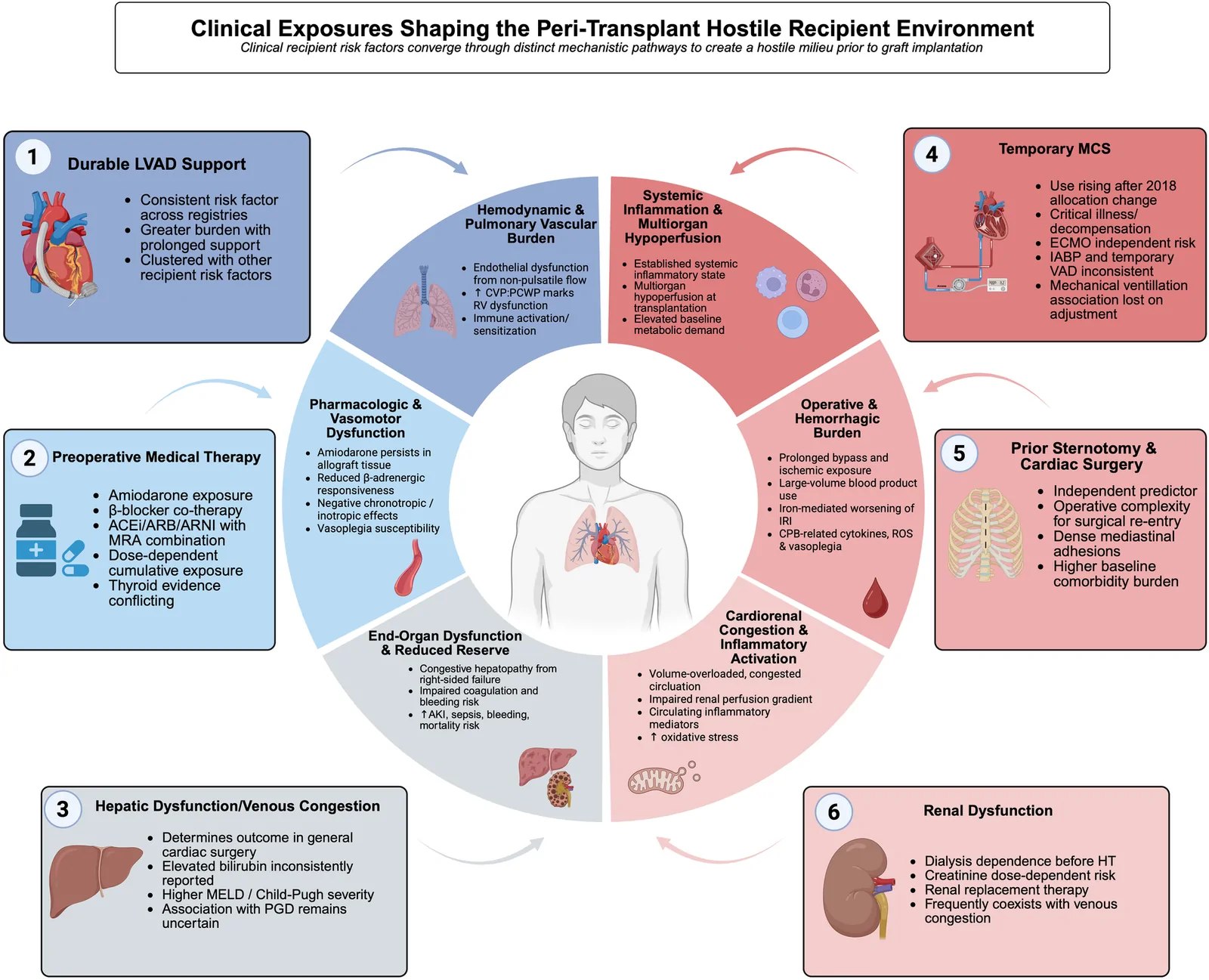
Clinical exposures that shape the peri-transplant recipient hostile environment. ACEi, angiotensin-converting enzyme inhibitor; AKI, acute kidney injury; ARB, angiotensin receptor blocker; ARNI, angiotensin receptor-neprilysin inhibitor; MELD, Model for End-Stage Liver Disease; MRA, mineralocorticoid receptor antagonist; HT, heart transplantation; CPB, cardiopulmonary bypass; ROS, reactive oxygen species; IRI, ischemia-reperfusion injury; ECMO, extracorporeal membrane oxygenation; IABP, intra-aortic balloon pump; VAD, ventricular assist device; CVP, central venous pressure; PCWP, pulmonary capillary wedge pressure;PGD, primary graft dysfunction; RV, right ventricle.

## 3 Mechanical circulatory support

### 3.1 Durable LVAD support

The need for durable left ventricular assist device (LVAD), in HT recipients has been well established across multiple registries and single-center analyses, as an independent recipient risk factor for the development of PGD (8, 15, 62–64). In a multi-center analysis from the International Consortium on PGD cohort of 4,125 HT recipients, severe PGD occurred in 12.7% of those supported with a durable LVAD versus 7.4% without (*P* < .001) (15). Likewise, a single-center cohort of 480 HT recipients reported that 80% of severe PGD patients were bridged with a continuous flow-LVAD (CF-LVAD) (OR, 3.86; 95% CI, 1.94-7.68; *P* < .001) (62); Moayedi et al. reported a similar finding with recipients supported by durable LVADs to be independently associated with severe PGD (OR 1.77; 95% CI, 1.13-2.77) (8). Duration of support is also relevant; among patients bridged with CF-LVADs, greater than one year of support was associated with severe PGD (OR, 2.48; 95% CI, 1.14–5.40; *P* = .022) (62). While the literature supports durable LVADs as a predictive risk factor, these patients often carry additional PGD risk factors, such as longer waitlist time, elevated pre-HT creatinine, elevated central venous pressure/pulmonary capillary wedge pressure (CVP/PCWP) ratio, and pre-HT amiodarone exposure (62). Additional exploration is thereby needed to establish a causative link.

The exact mechanisms that explain the contribution of durable LVADs to PGD, remains the subject of much investigation. Many studies have suggested an interplay between the effects of durable LVAD on RV function, inflammatory burden, and altered vascular physiology.

While a direct mechanistic link remains unknown, RV dysfunction and right-heart failure (RHF) have been associated with severe PGD across multiple cohorts (62, 65, 66). In Truby et al.'s analysis of 263 bridged-to-transplant patients with CF-LVAD, pre-HT hemodynamics among those with severe PGD showed higher CVP:PCWP ratios (0.70 ± 0.38 vs 0.54 ± 0.29; *P* = .004), which was driven by lower PCWP given the unchanged CVP between groups (*P* = .754), and lower pulmonary artery pressures (24.8 ± 9.1 vs 29.3 ± 10.2; *P* = .007), compared to those with no-to-moderate PGD, which the authors interpreted as suggestive of RV dysfunction (62). Takeda et al. reported severe PGD in 23.8% of LVAD-supported patients with late RHF compared to 6.67% in those without RHF (*P* = .012) (65); King et al. similarly reported post-LVAD RHF to be independently associated with a four-fold increased risk of PGD after multivariable adjustment (OR 3.91; 95% CI, 1.61–9.68; *P* = .003) (66).

The link between RHF or RV dysfunction in LVAD-supported patients and the development of PGD has been attributed to end-organ malperfusion, venous congestion, and a primed inflammatory response (65, 66). Both cohorts further note the possibility of a confounding factor: the reduced graft function may reflect that these recipients represent a sicker population at elevated risk of perioperative complications (65, 66). Importantly, the link between RV dysfunction and PGD remains unknown, and these findings should therefore be interpreted as hypothesis-generating.

LVAD triggers several immunological changes and inflammatory complications which have been associated with PGD development. The interactions between the implanted device and host immune system elicit an aberrant state of monocyte and T-cell activation, and cytokine upregulation, which promotes allosensitization to both class I and II HLA antibodies (67, 68). In a cohort of 132 patients supported by CF-LVADs, 23% demonstrated new allosensitization (69). As previously described, peak CPRA-LS for HLA-A is independently associated with increased severity of PGD (58); in a national registry analysis, PGD occurrence was higher among high class I (16.5% vs 10.6%; *P* = .01) and high class II (23.7% vs 9.2%; *P* < .01) PRA activity, although comparisons were unadjusted and the PGD definition used predates the ISHLT 2014 definition (70).

Moreover, despite the known effects of intraoperative blood product administration on allosensitization development (71), the direct association in the setting of LVAD implant remains elusive. Many studies allude to an inconsistent transfusion-dose or product-dependent relationship (72–74); a single-center cohort of VAD-bridged recipients with 93.5% receiving ≥ one red cell or platelet unit peri-implant found that the proportions of anti-HLA antibodies rose from 34.5% to 63.2% (*P* = .001), yet rates were comparable across recipients of red cells or platelet exposure (74). The strongest predictor of development of anti-HLA antibodies with LVAD implants, were patients with history of pre-existing allosensitization (OR, 6.3; *P* = .004) (74). Beyond HLA sensitization, device support elicits polyreactive IgG natural antibodies (Nabs) directed against apoptotic cells and oxidized epitopes. In a cohort of 206 HT recipients, pre-transplant IgG Nabs levels were significantly increased in VAD compared with non-VAD patients, and IgG reactivity to apoptotic cells was associated with the development of PGD in multivariable modelling (HR, 1.88; 95% CI, 1.23–2.87; *P* = .003) (75).

Additionally, the chronic non-pulsatile flow and supraphysiological shear stress of CF-LVAD have been proposed to contribute to endothelial dysfunction, dysregulated baroreceptor response, hemolysis, and von Willebrand factor degradation, ultimately leading to vascular pathology (76–79). CF-LVAD may induce structural arterial remodeling in the recipient's retained vasculature, with one study reporting an increase in aortic vascular stiffness from pre-implant to post-HT in patients bridged with non-pulsatile flow (2.8 ± 1.1 to 10.9 ± 4.7; *P* < .001) compared to non-bridged patients (3.4 ± 1.1; *P* < .001) (80). The vascular implications of LVADs appear to be substantial, however a gap remains between its link to the development of PGD.

### 3.2 Temporary MCS

In critically ill patients, temporary mechanical circulatory support (tMCS) offers a potential bridge to transplant. Recent studies show that the use of tMCS has grown sharply, likely attributable to the revised 2018 United Network for Organ Sharing (UNOS) donor heart allocation system (81, 82). In an era-based analysis from the International Consortium on PGD, Moayedi et al. report an increase in the proportion of patients supported on tMCS from 2010-2012 to 2019-2020 in pre-transplant intra-aortic balloon pump (IABP) (2.1% vs 20.9%; *P* < .001), pre-transplant Impella (0.2% vs 3.2%; *P* < .001), and pre-transplant peripheral ECMO (1.0% vs 2.9%; *P* = .068) (8). The use of these devices is associated with PGD, though not uniformly across all tMCS modalities. In the analysis by Cho et al., pre-transplant ECMO was an independent predictor of severe PGD (AOR, 2.55; 95% CI, 1.63–3.98; *P* < .001), whereas pre-transplant IABP and temporary VAD were not significant (*p* > 0.05) (9). In contrast, a single-center analysis of 508 HT recipients found pretransplant IABP to be independently associated with moderate/severe PGD (OR, 9.58; 95% CI,1.08–23.14; *P* = .045) (83). These findings should be compared cautiously as the endpoints differ in definition. Given the increasing use of tMCS and the inconsistent evidence regarding its association with PGD, the underlying mechanism behind this process warrants further investigation.

The mechanisms by which temporary support causes PGD remain less well characterized, and to our knowledge have not been tested in HT recipients. Recipients who require pre-transplant ECMO are often in cardiogenic shock, and those who require an IABP suffer significant hemodynamic instability, predisposing the body to marked systemic inflammation, high metabolic demands, and multiorgan hypoperfusion (84, 85). As such, recipients on tMCS represent some of the most critically ill patients, which may account for the elevated PGD rates, rather than a direct effect of the devices themselves.

Additionally, pre-operative mechanical ventilation is a consistently recognized recipient risk factor. The strength of this association has shown to be attenuated by adjustment across multiple contemporary studies. Among 570 HT recipients, preoperative ventilatory support was associated with moderate to severe PGD in the univariable analysis (OR, 4.34; 95% CI, 1.96–9.61; *P* < 0.001) but was not retained in the multivariable model (86). Likewise, a larger UNOS cohort reported no statistically significant difference of pretransplant ventilator use in severe PGD versus control in the univariable analysis (2.9% vs 2.2%; *P* = .37) (9). As such, mechanical ventilation may be best interpreted as a marker of critically ill, decompensated, and high-risk recipients, and is often concurrent with the use of temporary MCS, indirectly driving elevated PGD risk.

## 4 Preoperative medical therapy

Various medications have been implicated in the development of PGD, including amiodarone, renin-angiotensin-system (RAS) inhibitors, and a speculative contribution of thyroid hormone therapy. Interpretation of these pre-transplant drug risk factors is complicated by vasoplegia. The similarities between both entities make it difficult to delineate between the two, specifically in the context of preoperative medical therapy.

### 4.1 Preoperative amiodarone use

Amiodarone is a class III antiarrhythmic medication used to treat ventricular and atrial tachyarrhythmias, of which a significant proportion of patients with end-stage heart failure develop, by prolonging the myocardial refractory period (16, 87). Previous studies reported no associations with decreased survival (88) or impact on graft function (89, 90), none of which assessed PGD as an endpoint. More recently, in a single-center analysis of 183 patients, Dorsey et al. found no associations between amiodarone and severe PGD (*P* = .67) (91). In contrast, many studies report a strong association of amiodarone therapy with PGD: a 2026 meta-analysis identified recipient amiodarone therapy as the strongest severe PGD predictor (OR, 4.38; 95% CI, 2.48-7.75) (64); a dose-response analysis demonstrated an OR of 1.55 per 100 mg increase on the day of transplant and an OR of 1.67 per 18,300 mg increase in the 6-month cumulative dose (*P* < .001 for both) (17); an analysis of a 7,040 propensity-matched cohort found continued amiodarone therapy until HT to be associated with increased severe PGD (AOR, 1.63; 95% CI, 1.31-2.01) (16).

The exact mechanism by which amiodarone contributes to PGD is not completely known; however, many have been hypothesized. Given the drug's high lipophilicity, it is prone to a longer half-life of approximately 40-60 days, and thereby has been shown to remain at high concentrations in the post-HT period and may infiltrate the allograft tissue (92, 93). The presence of amiodarone in the allograft can exert its negative chronotropic and inotropic effects by calcium channel inhibition and *β*-receptor blockade (94, 95), ultimately, increasing post-transplant bradyarrhythmia's through nodal dysfunction (96), and impaired myocardial contractility, potentially explaining the contributions to PGD development (16).

Interestingly, Benck et al. identified that amiodarone alone was not associated with severe PGD (OR, 1.10; 95% CI, 0.43–2.85, *P* = 0.840), beta-blocker alone was (OR, 3.61; 95% CI, 1.48–8.81, *P* = .005), whereas the combination of amiodarone and *β*-blocker carried higher odds of severe PGD (OR, 12.14; 95% CI 5.03-29.29; *P* < .001) on single-variable analysis and remained independently associated after multivariable modelling (12). Although this mechanism is unknown, the authors speculate that this therapy exacerbates the sympathetic nervous system and *β*-adrenergic receptor dysfunction that has previously been described in DBD donors. The residual amiodarone and *β*-blockers then promote further dysfunction in the allograft with already compromised *β*-adrenergic receptors due to the dysregulated catecholamine storm in DBD donors (12).

### 4.2 RAS inhibition: ACEi, ARB, ARNI

The preoperative use of renin-angiotensin system (RAS) inhibitors in cardiac surgery is a well-known risk factor for postoperative acute kidney injury and mortality; however, its implications in HT are less characterized (97). RAS inhibitors such as ACEi, ARB, and ARNIs comprise key components of guideline-directed medical therapy which is frequently utilized for heart failure and critical in candidates awaiting HT (12). A novel finding by Benck et al. demonstrated that the use of ACEi/ARB/ARNI alone was not associated with severe PGD (OR, 1.45; 95% CI, 0.54–3.85; *P* = .459), likewise MRA alone was also null (OR, 1.63; 95% CI, 0.67–3.96; *P* = .282), whereas ACEi/ARB/ARNI plus MRA was (OR, 3.33; 95% CI, 1.57-7.08; *P* = .002) (12). While ACEi/ARB/ARNI + MRA treatment was identified as a recipient risk factor for severe PGD in Vervoorn's 2026 systematic review, it was absent from the pooled analysis which required a factor to be reported in ≥ 2 studies (64). Benck et al. hypothesized that ACEi/ARB/ARNI + MRA leads to the accumulation of vasodilatory peptides, notably bradykinin, which is known to increase during CPB (12, 98). Of note, bradykinin is a potent mediator of vasodilation rather than myocardial dysfunction; this pathway is therefore more consistent as a cause of vasoplegia rather than graft dysfunction (98, 99). This novel finding warrants further investigation given the prevalence of these medications among pre-HT patients.

### 4.3 Thyroid therapy

The relationship between thyroid therapy and PGD is multifaceted and complex, with evidence pointing in different directions depending on recipient and donor use. In a cohort of 151 HT recipients, an independent association was found between decreased free T4 levels and PGD (OR, 6.49; 95% CI, 2.26-18.61; *P* = .001) (100), a finding carried forward in Vervoorn's systematic review, that listed decreased free T4 levels as a recipient related PGD risk factor, though was not included for pooled analysis (64).

Thyroid hormone exerts cardioprotective effects, notably enhanced contractility through optimized calcium reuptake, and mitochondrial protection against IRI (101–103). While many of these mechanistic links are experimental model findings, supplementation in the recipient has been postulated to improve overall graft function post-transplant. Thyroid hormone therapy has historically been used as part of resuscitation protocols during procurement to aid in recruiting donor myocardium, a technique challenged by a contemporary trial reporting no benefit on transplanted hearts (104). Conflicting evidence, however, exists from multiple single-center studies challenging its use in recipients. In a cohort of 461 HT recipients, T3 supplementation was not associated with reduced incidence of PGD or mortality; however, the segmented time regression analysis revealed it may attenuate the severity of PGD (105). In contrast, among 266 HT recipients, those treated preoperatively with L-thyroxine had a lower risk for all-cause mortality (AHR, 0.24; 95% CI, 0.06-0.98; *P* = .047) (106). The associations between thyroid hormone therapy and PGD remain undefined; however, a randomized, double-blind, placebo-controlled trial is currently underway (NCT06428097) which may clarify this potential clinical benefit.

## 5 End-organ function

### 5.1 Renal dysfunction

Pre-transplant renal dysfunction is a consistently reported risk factor for PGD. Pre-HT creatinine elevations and preoperative dialysis dependence have been reported across single-center and multicenter cohorts (8, 15, 62, 86, 107). Of these, the cohort of 2,746 patients reported by Moayedi et al. identified renal replacement therapy at the time of HT as a clinical predictor of severe PGD (OR, 2.41; 95% CI, 1.31–4.43) (8). Moreover, a dose-dependent relationship in recipient creatinine with an OR of 3.68 per increase of 1 mg/dL was associated with PGD risk (95% CI, 2.08-6.50; high certainty) (4).

Recipient renal dysfunction as a contributor to PGD is best explained as a combination of insults rather than a single mechanism and may be best understood as a marker of cardiorenal syndrome (108). Pre-transplant renal dysfunction, in part, may reflect RV dysfunction and venous congestion, where elevated right-sided pressures retrogradely propagate and impair the renal perfusion gradient (62, 109). Consistent with this, Truby et al. describe elevated pre-transplant creatinine levels and CVP/PCWP ratio as surrogates for venous congestion (15). Additionally, inflammatory mediators and oxidative stress associated with renal insufficiency may contribute to graft dysfunction, in keeping with a type III cardiorenal syndrome (62, 110). Whether renal dysfunction contributes directly to graft injury, to our knowledge, has not been examined.

### 5.2 Hepatic dysfunction

Hepatic dysfunction is a well-known predictor of overall outcome in cardiac surgery. In a pooled analysis of 48,891 patients undergoing cardiac surgery, those in the higher score group (elevated MELD or Child-Turcotte-Pugh scores) had higher perioperative mortality odds (OR 3.72, 95% CI 2.75–5.03) along with increased odds of adverse events, namely AKI, neurologic events, prolonged ventilation, sepsis, and bleeding/transfusion, compared to patients in lower score groups (111). The reported association of hepatic dysfunction and PGD, in contrast, is not as clear. Elevated bilirubin, a common marker for hepatic congestion, has been inconsistently associated with severe PGD. Elevated bilirubin has been associated with PGD in unadjusted modelling of a single-center cohort of 570 recipients (OR, 1.11; 95% CI, 1.02–1.21; *P* = .012), yet was not significant following multivariable modelling (86). Large multicenter cohorts also found this association to be null (8, 9, 15). Therefore, recipient hepatic dysfunction remains an unconfirmed predictor of PGD.

## 6 Operative factors

At the time of surgery, several recipient-attributable risks, namely prior sternotomy, have been identified as predictors of PGD. While intraoperative blood product administration and CPB time are not inherent recipient characteristics and more in keeping with perioperative exposures, their magnitude is largely determined by underlying recipient variables and are therefore considered below.

### 6.1 Prior sternotomy and cardiac surgery

Across single-center, national, and meta-analytical data, prior sternotomy is a consistently described recipient surgical risk factor. Namely, in the largest recent UNOS cohort of 5,097 recipients, prior sternotomy was an independent predictor of severe PGD (AOR, 1.83; 95% CI, 1.33–2.51; *P* < .001) (9). Moreover, in a single-center analysis of 255 recipients, recipients with prior sternotomy (55%) exhibited higher rates of mild to severe PGD (32% vs 18%; *P* = .015) and remained an independent predictor after multivariable modelling (OR, 2.71; 95% CI,1.36–5.42; *P* = .0047) (14). While prior sternotomy may in part be a composite proxy for LVAD-implanted patients, a recent meta-analysis found non-LVAD sternotomy as an independent risk factor (OR, 1.96; 95% CI, 1.01-3.81) (64). Nonetheless, recipients with prior sternotomy differ at baseline being of older age, increased rates of ischemic cardiomyopathy, higher body mass index, and longer waitlist times (14).

Reoperating on a recipient with prior sternotomy remains technically difficult as the sternum may have several adhesions to vital cardiac structures (112). Consequently, these procedures tend to be associated with longer CPB and ischemic time, higher rates of bleeding, and increased use of transfusion products (113–115). These factors are well-known predictors of PGD, as discussed below; therefore, the operative complexity that comes with prior sternotomy may indirectly act on PGD.

### 6.2 Perioperative exposures: intraoperative blood administration & CPB

Intraoperative transfusion has an established association with PGD, and its use largely reflects recipient characteristics; whether that be candidates supported by LVADs, recipients with prior sternotomy requiring extensive adhesiolysis, or those who are anemic at baseline (14, 64, 78). In a single-center cohort of 734 recipients, each blood product was associated with severe PGD risk but not mild-moderate PGD risk; packed red blood cells (OR, 1.18; 95% CI, 1.12-1.25; *P* < .001), platelets (OR, 1.45; 95% CI, 1.24-1.70; *P* < .001), plasma (OR, 1.13; 95% CI, 1.07-1.19; *P* < .001), and cryoprecipitate (OR, 1.04; 95% CI, 1.02-1.07; *P* = .002) (12). Likewise, blood product administration was associated with severe PGD across multiple studies but could not be pooled for analysis due to definition heterogeneity (64). However, the relationship is largely associative as the mechanistic link is hypothesized and remains unproven. Proposed pathways include volume overloading impeding RV function (12), inflammatory mediators that contribute to allograft injury (116, 117), or the release of iron which worsens the ischemia-reperfusion injury (118). Benck et al. further speculate that intraoperative bleeding itself may lead to hypotension or ischemia which directly affects the allograft; if identifiable, however, it should be explicitly defined as secondary graft dysfunction rather than PGD (12). Importantly, the supporting evidence for these mechanistic pathways is largely extrapolated from general cardiac surgery and transfusion medicine rather than transplant-specific contexts.

Prolonged CPB time has been associated with the development of PGD (6, 64); namely, a recent European cohort found longer extracorporeal circulation (equivalent to CPB in this context) was one of four independent predictors of PGD with an AOR of 1.75 per 60-minute increase (95% CI, 1.44–2.14; *P* < .0001) (13). This association may be explained by the extensive interaction with the synthetic CPB circuit which exposes the recipient to a systemic inflammatory state characterized by cytokine release, formation of oxygen-free radicals, and dysfunction in the coagulation cascade (119). Further, the propagation of these mediators, namely C5a, E-selectin, and nitric oxide synthase, promote the loss of vascular tone, leading to vasoplegia (120). Despite the association, these intraoperative factors may be an effect of PGD rather than a cause (6). Truby et al. challenge the inclusion of CPB time in PREDICTA score, stating that it may be a consequence of moderate-to-severe PGD rather than a predictor of it (50). Likewise, Steffen et al. noted that longer CPB time within their cohort reflected the need for MCS during weaning (121). As such, these associations must be interpreted with caution, as directionality cannot be established from observational data and may represent reverse causation.

## 7 Future directions & evidence gaps

There remains no interventional study that examines modifying recipient risk factors and its direct influence on reducing rates of PGD. The current evidence base and mechanistic findings are extrapolated from observational and retrospective studies which establish association rather than causation. Importantly, this precludes distinguishing whether these reported risk factors contribute directly to graft injury or reflect the severity of illness at transplantation, Table 1. Further translational and interventional studies that investigate the pathophysiology of PGD, and specifically establish the mechanism of action for each recipient risk factor, are a priority. Moreover, with the increasing deterioration of the HT recipient population, studies examining the overall impact of frailty on the development of PGD are warranted, as frailty serves as an important prognostic marker for HT eligibility and is associated with significant mortality rates (122). Until such evidence is available, the recipient risk factors identified by the literature should be used to guide recipient optimization.

Beyond recipient optimization, PGD must also be recognized as a broad umbrella term encompassing phenotypes that differ by ventricular involvement, severity, and trajectory of recoverability. This heterogeneity argues against a uniform approach to prevention, prediction, and management of PGD. Personalizing treatment begins with preoperative prediction. Existing prediction models carry limitations: the ABCE score lacks external validation (12); the RADIAL score, although the most validated tool, is poorly predictive in contemporary cohorts (8, 123); and the PREDICTA score relies on intraoperative variables limiting its utility (124). Machine learning-based models developed from the International Consortium on PGD have been validated, although they do not consider DCD hearts (125–127). Likewise, the management of PGD should be individualized to the clinical phenotype at hand. A contemporary review proposed a personalized algorithm composed of three phases: a preoperative phase applying validated prediction tools to guide optimal allocation and preservation strategies, an intraoperative phase using intraoperative markers to enable early detection and management, and a postoperative phase assessing graft recoverability to guide care (7).

## 8 Conclusions

Primary graft dysfunction remains the leading cause of early mortality and morbidity following heart transplantation. Key recipient risk factors discussed in this review, namely amiodarone exposure, durable LVAD support, pre-transplant ECMO, prior sternotomy, and renal dysfunction, have emerged as consistently reported risk factors of severe PGD. The association of temporary mechanical circulatory support with severe PGD, however, is not uniform across devices, with findings for temporary VAD and IABP remaining inconsistent. In an era where donor preservation technology is advancing and the donor pool is expanding, there remains a substantial need to optimize recipients prior to heart transplant, in turn improving outcomes and minimizing PGD. Optimizing recipients begins with understanding the mechanisms by which recipient factors contribute to PGD.

## Glossary

ACEi: angiotensin-converting enzyme inhibitor
AKI: acute kidney injury
ARB: angiotensin receptor blocker
ARNI: angiotensin receptor-neprilysin inhibitor
ATP: adenosine triphosphate
AWIT: asystolic warm ischemic time
BiVAD: biventricular assist device
CF-LVAD: continuous-flow left ventricular assist device
CLEC4C: C-type lectin domain family 4 member C
CPB: cardiopulmonary bypass
CPP: cerebral perfusion pressure
CPRA-LS: calculated panel reactive antibody, locus-specific
CVP: central venous pressure
DBD: donation after brain death
DCD: donation after circulatory death
DO_2_i: indexed oxygen delivery
ECMO: extracorporeal membrane oxygenation
EGD: early graft dysfunction
HLA: human leukocyte antigen
HT: heart transplantation
IABP: intra-aortic balloon pump
ICP: intracranial pressure
IL: interleukin
IMPACT: Index for Mortality Prediction After Cardiac Transplantation
IRI: ischemia-reperfusion injury
ISHLT: International Society for Heart and Lung Transplantation
KLKB1: plasma kallikrein
LV: left ventricle
LVAD: left ventricular assist device
MCS: mechanical circulatory support
MELD: Model for End-Stage Liver Disease
MRA: mineralocorticoid receptor antagonist
mtDNA: mitochondrial DNA
Nabs: natural antibodies
O_2_ER: oxygen extraction ratio
PCWP: pulmonary capillary wedge pressure
pDC: plasmacytoid dendritic cell
PGD: primary graft dysfunction
PRA: panel reactive antibody
RAS: renin-angiotensin system
RHF: right-heart failure
RV: right ventricle
SVR: systemic vascular resistance
tMCS: temporary mechanical circulatory support
TNF: tumour necrosis factor
UNOS: United Network for Organ Sharing
VA-ECMO: veno-arterial extracorporeal membrane oxygenation
VAD: ventricular assist device
VO_2_i: indexed oxygen consumption.
